# Analysis of Decision Tree and K-Nearest Neighbor Algorithm in the Classification of Breast Cancer

**DOI:** 10.31557/APJCP.2019.20.12.3777

**Published:** 2019

**Authors:** Harikumar Rajaguru, Sannasi Chakravarthy S R

**Affiliations:** *Department of Electronics and Communication Engineering, Bannari Amman Institute of Technology, India. *

**Keywords:** Breast cancer, mammogram, KNN, PCA, decision tree

## Abstract

**Objective:**

The death rate of breast tumour is falling as there is progress in its research area. However, it is the most common disease among women. It is a great challenge in designing a machine learning model to evaluate the performance of the classification of breast tumour.

**Methods:**

Implementing an efficient classification methodology will support in resolving the complications in analyzing breast cancer. This proposed model employs two machine learning (ML) algorithms for the categorization of breast tumour; Decision Tree and K-Nearest Neighbour (KNN) algorithm is used for the breast tumour classification.

**Result:**

This classification includes the two levels of disease as benign or malignant. These two machine learning algorithms are verified using the Wisconsin Diagnostic Breast Cancer (WDBC) dataset after feature selection using Principal Component Analysis (PCA). The comparison of these two ML algorithms is done using the standard performance metrics.

**Conclusion:**

The comparative analysis results indicate that the KNN classifier outperforms the result of the decision-tree classifier in the breast cancer classification.

## Introduction

 Breast cancer refers to a disease which is found more among the women in developed and in developing nations. The statistical report of World Health Organization proves that the existence of breast tumour is high for women in developed countries. Globally, there are many statistical reports are published to define the brutality of breast tumour. The curing of breast cancer at prior phase is feasible and it will reduce the death rate of breast cancer. Thus to minimize the death rate, earlier discovery and precise analysis is more noteworthy. Analysis of diseases using medical imaging is more prominent in the medical field (Al-Hajj et al., 2003). Regular screening of breast is required by the doctors for the earlier discovery of the disease. This earlier discovery of breast cancer denotes the identification of disease before the symptoms are felt by the patients. This denotes the observation of tissues in the breast-part of the patients for any abnormal lumps. At this stage, a fine-needle aspiration (FNA) biopsy procedure is needed if any lump or mass is appeared on the breast while screening (Sannasi Chakravarthy et al., 2019). This lump is termed as tumour. This biopsy method is used to collect a few cell samples in the region of lump occurrence in breast. It is a simple procedure and it does not require any hospitalization for the patients. This procedure is commonly followed one for the screening of breast cancer because of its accuracy. Then the microscopic examination of these samples is done by the physicians to decide that whether it is a tumour or not (Rajaguru et al., 2019). The tumour is categorized as benign or malignant. 

In general, the tumour flinches in the tissues of breast and it blowout to lymph area which is present in the armpit part of the body. After this, the tumour cells can now transportable and will affect the adjacent parts (liver) of the body. This will lead to the chance of formation of tumours in the adjacent parts of the body. A tumour cell is said to be benign if it is not harmful and malignant tumour is said to be harmful. Benign tumour may certainly not require any diagnosis but not malignant tumour type. Malignant type tumours can able to harm the native parts by blow-out the enzymes.

**Figure 1:**
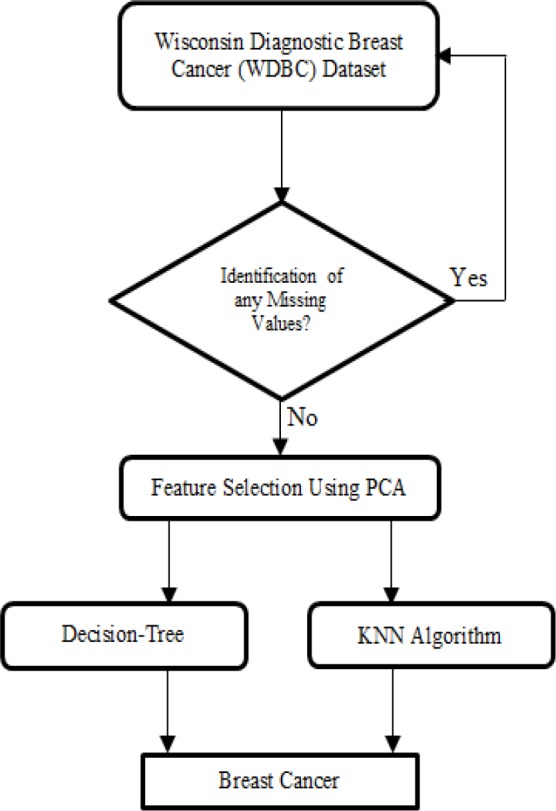
Proposed Method

More machine learning algorithms are applied for building a classification model for the standard WDBC input dataset. But it is difficult to produce a comprehensive model for the problem of breast cancer classification because it is very much non-linear. This study intends to suggest a machine learning based classifiers to obtain an efficient classification model for the classification problem of breast tumour. The Figure 1 illustrates the work plan of the proposed work. The WDBC dataset is taken as the input and check for any missing values. If any missing values found, then pre-processing is required in the taken dataset. If there is no missing values, then PCA based feature selection is made. These features are utilized by the Decision-Tree algorithm and KNN algorithm to perform the breast cancer classification.

## Materials and Methods

*A. Dataset*

The analysis of classifiers used in the work are tested with the Wisconsin Diagnostic Breast Cancer (WDBC) dataset as available in the UCI repository. There are about 569 instances with 32 features are present in the WDBC dataset. Out of 32 features, the work utilizes the diagnosis (M denotes malignant and B denoted Benign) feature for classification and the remaining features like radius_mean, texture_mean, perimeter_mean, area_mean, smoothness_mean, compactness_mean, concavity_mean, concave points_mean, symmetry_mean, fractal_dimension_mean, radius_se, texture_se, perimeter_se, area_se, smoothness_se, compactness_se, concavity_se, concave points_se, symmetry_se, fractal_dimension_se, radius_worst, texture_worst, perimeter_worst, area_worst, smoothness_worst, compactness_worst, concavity_worst, concave_points_worst, symmetry_worst and fractal_dimesnsion_worst. And the attribute id refers to the ID number of the patients’ sample (Dua and Karra Taniskidou, 2017). 

**Figure 2:**
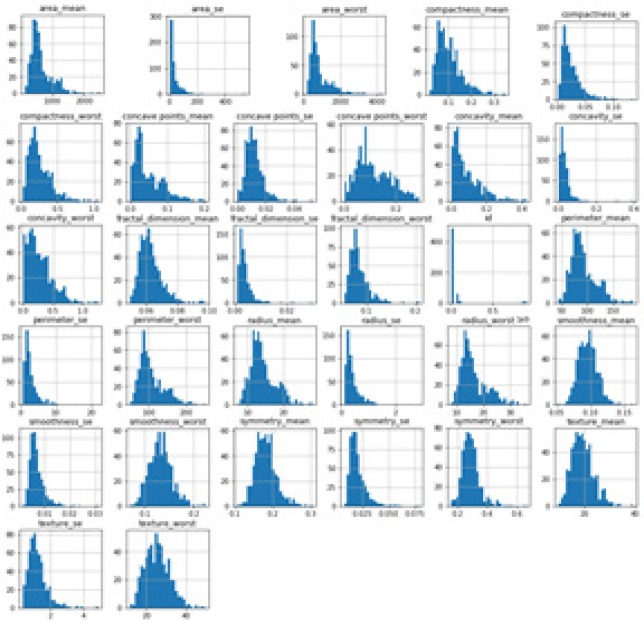
Histogram Plot of Each Attribute

Ten real-valued attributes along with mean, se (standard error) and worst obtained for each cell nucleus. These 30 features are worked-out from the digitally acquired image of FNA of a breast lump or mass. They refer to the properties of cell nuclei exists in the digitized image of FN and all these attributes are obtained with 4 significant digits. The 569 instances has the class distribution of 357 benign and 212 malignant samples (Dua and Karra Taniskidou, 2017). Since it does not contains any missing values, it is taken as such for the work. Figure 2 shows the histogram plot of each features present in the input dataset.

*B. PCA for Feature Selection*

As given in the Figure 2, the input attributes are huge; this implies the requirement of feature selection to improve the speed and accuracy of the ML algorithms used for classification. If the feature space used is very large, then we may go for feature selection and so it is termed as dimensionality reduction of input features available in the dataset. A simple and widely used technique for the problem of two-class classification is Principal Component Analysis (PCA). The usage of PCA is to recognize the ways of peak disparity in the applied dataset (wold et al., 1987). Thus by using PCA, a huge dataset is reduced with less number of significant features. PCA technique for feature selection is applied to both training and testing features. The primary task of PCA is to identify patterns in the input dataset to detect any resemblance and variances among the distinct attributes present (Abdi and Williams, 2010).

Applying a PCA is fair calculating the Eigen values (λ_i_) and Eigen vectors (E_i_) of the input’s correlation matrix as: 

Σ=* Z*^T^* Z*                                                  (1)

where 

*Z= Y-μ*_y_                                                  (2)

and Y denotes an A X B matrix, here each row refers one of A trials with each column refers one of B features and *μ*_y_ denotes the value of empirical mean of the input. Finally the principal components are defined as: 

*P=Z.V* ^T^                                                  (3)

If there is a need for first s components, then it adapts to product the input with just the first s rows of *V* (right singular-vector) (Gu et al., 2018).

*C. Classification using Decision Tree Algorithm*

Decision-Tree algorithm is one of the most widely used ML algorithm for the problems of both regression and classification. In general, the algorithm imitates like the thinking of humans making it as so popular and easier to interpret the inputs with better understanding of problem statement (Olanow et al., 2001). In this algorithm, a decision-tree denotes a tree with its node refers to the attribute whereas its link refers to a decision rule and its leaf node refers to an output class. The aim is to produce a tree-like structure for the input features and creates a unique output at every leaf ( Pandya and Pandya, 2015).

Our problem involves binary class classification, we used ID3 algorithm (Dai et al., 2016) for building the decision-tree. The necessity of adopting the Decision-Tree algorithm in our problem is to build a training model that should be utilized for the prediction of output class by means of inferring the decision-rules understood from the earlier trained data.

The pseudocode for Decision-Tree algorithm is as follows:

i. Keep the best feature of the input attributes at the root portion of the tree.

ii. Then make a splitting of training dataset into subsections. 

iii. These splitted subsets can be done by making the each subset with data having the similar value for a input attribute.

iv. Now repeat the step 1, 2 and step 3 on each subset till the leaf portion in every branches of the tree is found.

While using decision-tree algorithm for classification, the entire input training data is taken as root. The depth analysis of decision-tree algorithm is also considered while using it for the classification problem. If the depth of the algorithm is higher, then the problem of overfitting will occurs (Tsang et al., 2009). The size represents the quantity of nodes present in the tree. Since each node of decision tree algorithm is used to make a binary classification, the size should be as large as 2^d+1^-1, where d indicates depth of the tree. The optimal depth used for the algorithm in the work is 3. And this optimal depth can be applied to any binary classification problem. For higher depth, the problem of over-fitting takes place. And for multi-class classification problems, the depth may varies. 

*D. Classification using KNN Algorithm*

KNN algorithm is a non-parametric approach used for the problem for classification. The approach customs the information about its neighbour points for the classification of output labels (Dudani, 1978). In 1951, the KNN algorithm is introduced by Fix and Hodges for the application of classification problems (Saini et al., 2013). KNN algorithm is used widely for both pattern recognition and classification applications. KNN has the capability to predict the target class with more precise in simpler manner. KNN is simply belongs to the type of lazy-learning algorithm with its function is estimated only locally and the entire calculation is overdue till the process of classification (Mejdoub and Amar, 2013).

The KNN algorithm used in our problem, considers the output as a target class. The problem is solved or classified by the majority voting of its neighbours, where the value of K is taken as a small and real valued positive integer (Cunningham and Delany, 2007). The KNN algorithm is more sensitive to the local part of the input data which makes it more unique to the classification problem.

The pseudocode for KNN algorithm is follows,

i. Calculate the Euclidean distance d(*x,x*_i_) where *i* =1, 2 . . . n between the points.

ii. Sort the above obtained n distances in ascending order.

iii. Consider *k* as a real-valued positive integer and the first *k* distances are taken from the above arrangement.

iv. Now by using these k distances, estimate the *k *points.

v. For *k*≥0,*k*_i_ is the amount of points correspond to the i^th^ category amid the *k* points.

vi. Check the condition of* k*_i_*>k*_j_ if and if the value of i not equal to j, the keep *x *in the category *i*.

The value of *k* is taken based up on the input data; if the value of *k* is larger enough, then it will reduce the effect of noise on the output class. The optimal value of *k* chosen for the problem is 13 and increasing over this value results in under-fitting. The presence of irrelevant features (noise) in the input dataset will reduce the accuracy or precision of KNN algorithm even with its high efficiency of classification (Frigui and Gader, 2008). Thus feature selection and pre-processing is important for the proposed work.

**Table 1:** Performance Comparison of ML Algorithms

ML Algorithms	Comparison Metrics				
	Sensitivity	Specificity	Accuracy	Precision	Matthews Correlation Coefficient
Decision-Tree Algorithm	92	89.74	91.23	94.52	80.81
KNN Algorithm	95.95	95	95.61	97.26	90.44

**Figure 3.**
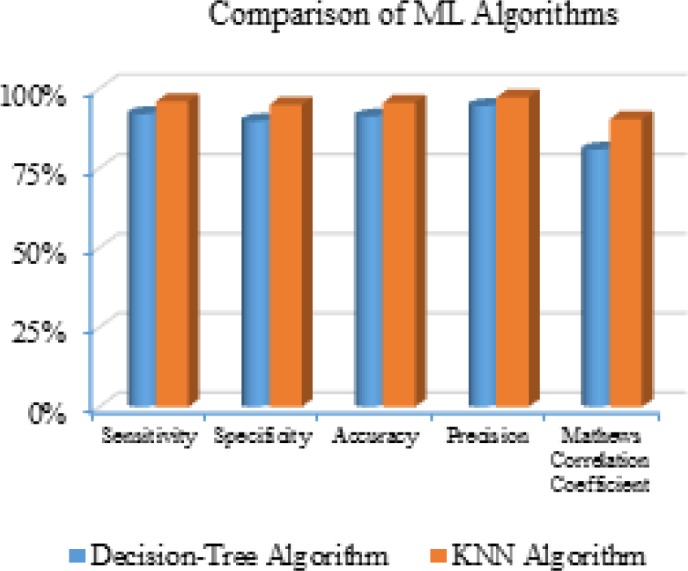
Performance Comparison

**Table 2:** Comparison of Proposed Model

References	Methodology	Classification Accuracy (%)
Srivastava et al., 2014	Hybrid feature vectors with k-nearest neighbour as classifier	87
Saini et al., 2015	Texture feature vectors with artificial neural network as classifier.	87.5
Pawar et al., 2016	Wavelet based feature vectors with genetic fuzzy model.	89.47
Gardezi et al., 2016	Curvelet coefficient based grey level co-occurrence matrix and curvelet coefficient based geometric invariant shift method with support vector machine as classifier.	92.39
Vaidehi et al., 2015	Texture feature vectors with sparse representation model.	93.75
Harefa et al., 2017	Texture feature vectors with support vector machine as classifier.	93.88
Pratiwi et al., 2015	Texture feature vectors with radial basis function as classifier.	93.98
Bazila Banu A et al., 2018	Gradient boosting model as a classifier	94.11
Proposed work	PCA with K-NN algorithm as classifier	95.95

## Results

The results of decision-tree and KNN algorithm as classifier are related using the standard metrics of classification. Sensitivity refers to the possibility that a problem will categorize ‘malignant’ among the input data with the malignant and Specificity refers to the portion of benign with non-benign category (Abirami et al., 2016). Accuracy denotes the typical mean of these above two metrics. Accuracy denotes the amount of appropriately categorized data whereas the Precision denotes the amount of malignant that are malignant (Sokolova and Lapalme, 2009). The Matthews Correlation Coefficient is a measure calculated between the actual and obtained output category in the work.

The Table 1 shows the performance comparison of machine-learning algorithms used for the breast cancer classification and its graphical analysis is shown in the Figure 3. From the above Table and graph, the results are improved for both the algorithms due to the appropriate feature selection by PCA approach. And in terms of metrics used, KNN classifier is the competent ML algorithm when compared with the Decision-Tree classifier. This is owing to the point that the KNN as a classifier is more sensitive to the local input patterns; this facilitates the KNN classifier to perform well when compared with the Decision-Tree classifier in the proposed work. 

## Discussion

The comparison of accuracy metric of the proposed work is done with the related works as in Table 2. 

The aim of the work is to create a machine-learning model for the efficient classification of breast cancer. For this, the study utilizes a tree-based and non-parametric type machine learning algorithms for the classification problems. Using PCA technique for the feature selection on the UCI Wisconsin Breast Cancer Diagnosis dataset outcomes a noteworthy impact headed for outlining the variance in input dataset and for the binary classification. Thus the proposed model using decision tree and KNN algorithm is implemented after feature selection which shows that the lazy learning algorithm is more effective than the decision-tree algorithm. The future work of the paper is to propose a dynamic and multi-class recognition for the detection of breast cancer using different feature selection methods with different datasets.
